# Vom Lebensmittel zur Substanz menschlichen Ursprungs – Die Verordnung (EU) 2024/1938 und ihre Auswirkungen auf den Umgang mit humaner Milch

**DOI:** 10.1055/a-2816-5471

**Published:** 2026-03-24

**Authors:** Daniel Klotz, Janina Hahnloser, Dagmar Schilling-Leiß, Manfred Doll, Monika Berns

**Affiliations:** 1 Abteilung für Neonatologie und Pädiatrische Intensivmedizin, Kinderzentrum, Evangelisches Klinikum Bethel, Universitätsklinikum Ostwestfalen-Lippe, Bielefeld, Germany; 2 Frauenmilchbank-Initiative e. V., Nürnberg, Germany; 3 27656 Referat 113 Blut, Blutprodukte, Sera, Impfstoffe und Gewebe, Bundesministerium für Gesundheit, Bonn, Germany; 4 Paul-Ehrlich-Institut, Langen, Germany; 5 14903 Klinik für Neonatologie, Charité Universitätsmedizin Berlin, Berlin, Germany; 6 Max Rubner-Institut (MRI), Bundesforschungsinstitut für Ernährung und Lebensmittel, Nationale Stillkommission, Karlsruhe, Germany

**Keywords:** Frauenmilch, Humanmilch, Regulierung, SoHO, Substanz menschlichen Ursprungs, donor human milk, human milk, regulation, SoHO, substance of human origin

## Abstract

Die Verordnung (EU) 2024/1938 über Qualitäts- und Sicherheitsstandards für zur Verwendung beim Menschen bestimmte Substanzen menschlichen Ursprungs ( *Substances of Human Origin* , SoHO-Verordnung) stellt einen Paradigmenwechsel in der rechtlichen Einstufung von Humanmilch dar. Dieser Übersichtsartikel zeigt die Neuerungen gegenüber der bisherigen Regulierung humaner Milch in der Europäischen Union auf, gibt den Stand der nationalen Umsetzung der SoHO-Verordnung sowie europäischer Harmonisierungsbestrebungen wieder und fasst die möglichen Auswirkungen der Verordnung auf den klinischen Umgang mit humaner Milch zusammen.Insgesamt ergeben sich neue Anforderungen für den Umgang mit humaner Milch, insbesondere hinsichtlich einer Registrierungspflicht für alle Einrichtungen, die eine Tätigkeit mit einem SoHO ausüben, und einer Erlaubnispflicht für bestimmte SoHO-Einrichtungen (SoHO-Betriebsstätten).Zu beachten ist, dass die Verordnung (EU) 2024/1938 sich nicht nur auf den Umgang mit gespendeter Humanmilch, sondern auch auf den Umgang mit verarbeiteter Muttermilch auswirkt.Eine gestaffelte Übergangsfrist bis August 2028 ermöglicht eine schrittweise Implementierung unter Berücksichtigung europäischer Harmonisierungsbestrebungen und nationaler Herausforderungen.ABSTRACTRegulation (EU) 2024/1938 on quality and safety standards for substances of human origin intended for human application (Substances of Human Origin, SoHO Regulation) represents a paradigm shift in the legal classification of human milk. This review article presents the innovations compared to previous regulations of human milk, describes current European harmonization efforts as well as the state of national implementation, and summarizes the possible impacts of the regulation on the clinical handling of human milk. Overall, new requirements arise for the handling of human milk, particularly with regard to an obligation for registration of entities that carry out a SoHO activity and for authorization as a SoHO establishment.It should be noted that Regulation (EU) 2024/1938 applies not only to the handling of donor human milk but also to the handling of processed mother’s own milk.A transition phase until August 2028 enables gradual implementation while taking into account European harmonization efforts and national challenges.

## Hintergrund

Die rechtliche Einstufung humaner Milch in den Mitgliedstaaten der Europäischen Union (EU) war bis zum Inkrafttreten der SoHO-Verordnung ( *Substance(s) of Human Origin* , SoHO) ausgesprochen heterogen. In den meisten Mitgliedsstaaten lag keine spezifische Klassifikation und Regulierung humaner Milch vor 1 .

Aufgrund dieser Regulierungslücke nahm die Europäische Kommission Humanmilch ausdrücklich in die geplante Novellierung des Rechtsrahmens für Blut, Gewebe und Zellen auf 2 .

Nach einem mehrjährigen Konsultations- und Verhandlungsprozess 3 4 5 wurde die Verordnung (EU) 2024/1938, die sogenannte SoHO-Verordnung, am 13. Juni 2024 verabschiedet und trat am 6. August 2024 in Kraft 6 . Ab dem 7. August 2027 wird der Großteil der Verordnung unmittelbar in den EU-Mitgliedstaaten Anwendung finden.

## Ziele der Regulierung

Die zentralen Ziele der neuen SoHO-Verordnung sind die Erhöhung der Sicherheit für SoHO-Spender und SoHO-Empfänger sowie die Verbesserung der Verfügbarkeit von SoHO innerhalb der EU. Indem die EU-Gesetzgebung neben Blut, Geweben und Zellen erstmals auch auf Darmmikrobiota und Humanmilch Anwendung findet, werden bestehende Regelungslücken geschlossen. Durch Angleichung der Rechtssysteme sowie der Qualitäts- und Sicherheitsstandards in den EU-Mitgliedstaaten soll ein grenzüberschreitender Austausch erleichtert werden. Zudem werden erstmals alle Einrichtungen erfasst, die Tätigkeiten mit SoHO ausüben, einschließlich solcher, die ausschließlich mit der Anwendung von SoHO befasst sind 6 .

## Anwendungsbereich der SoHO-Verordnung

Die SoHO-Verordnung definiert Humanmilch grundsätzlich als Substanz menschlichen Ursprungs und beendet damit ihre bisherige Klassifikation als Lebensmittel 7 .

Die Verordnung findet grundsätzlich Anwendung bei gespendeter Humanmilch (Frauenmilch). Sie findet ebenfalls Anwendung bei Humanmilch für das eigene Kind (Muttermilch), sofern die Muttermilch in einer SoHO-Einrichtung verarbeitet, insbesondere, wenn sie pasteurisiert wird ( Tab. 1 ).

**Table:** **Tab. 1** Begriffsdefinitionen Humanmilch, Muttermilch, Frauenmilch (nach 8 ).

Begriff	Definition	Kommentar
Humane Milch (HM)	Milch menschlichen Ursprungs	Englische Bezeichnung: human milk (HM), expressed breast milk (EBM)
Muttermilch (MM)	Milch einer laktierenden Frau für ihr Kind	Englische Bezeichnungen: mother’s own milk (MOM), maternal milk (MM)
Frauenmilch (FM)	Gespendete humane Milch zur Ernährung eines anderen Kindes als des eigenen der Spenderin	Synonyme: Donormilch, Spendemilch, Spenderinnenmilch
		Englische Bezeichnung: donor human milk (DHM), donor milk (DM)

Keine Anwendung findet die Verordnung bei Muttermilch, sofern sie ausschließlich zur Ernährung des eigenen Kindes ohne Verarbeitung durch eine SoHO-Einrichtung verwendet wird.

Ebenfalls nicht erfasst werden Tätigkeiten in einem persönlichen Umfeld, wie das Stillen oder die Abgabe von Humanmilch an Kinder von Freunden oder Verwandten, solange keine kommerzielle Gewinnabsicht vorliegt und diese Tätigkeiten nicht mit einer gewerbsmäßigen Regelmäßigkeit ausgeübt werden.

## Definition von SoHO-Einrichtungen und SoHO-Betriebsstätten

**Abb. 1:**
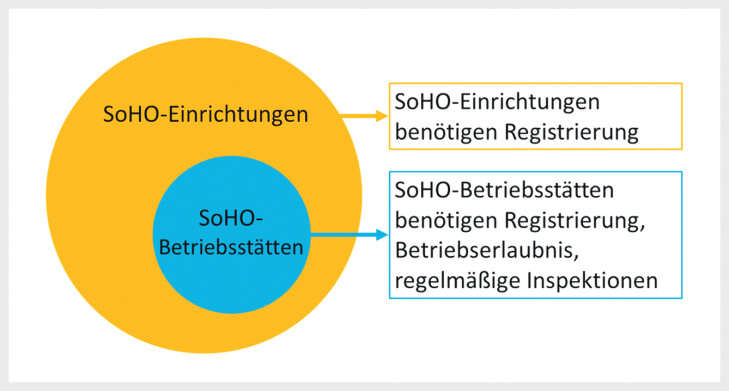
Unterscheidung zwischen SoHO-Einrichtungen und SoHO-Betriebsstätten.

Die Verordnung unterscheidet zwischen SoHO-Einrichtungen und SoHO-Betriebsstätten als Unterform der SoHO-Einrichtung ( Abb. 1 ).

SoHO-Einrichtungen sind alle Einrichtungen, die zumindest eine SoHO-Tätigkeit ausüben, die von der Spenderregistrierung bis zur Anwendung von SoHO-Präparaten beim Menschen reichen können. SoHO-Einrichtungen werden sich vor Aufnahme ihrer Tätigkeit auf einer sich noch in der Entwicklung befindlichen Online-SoHO-Plattform der EU-Kommission registrieren müssen.

SoHO-Betriebsstätten hingegen sind SoHO-Einrichtungen, in denen mindestens eine der folgenden Tätigkeiten bzw. Tätigkeitskombinationen erfolgt: Verarbeitung *und* Lagerung von SoHO oder die Freigabe von SoHO. Besonders die Freigabe der SoHO zur Anwendung am Menschen ist ein kritischer Verfahrensschritt, bei dem geprüft wird, ob eine SoHO die festgelegten Qualitäts- und Sicherheitskriterien erfüllt. SoHO-Betriebsstätten üben damit bestimmte als risikoreich klassifizierte Tätigkeiten mit SoHO aus und müssen daher mehr Pflichten erfüllen als reine SoHO-Einrichtungen, die diese oben genannten Tätigkeiten nicht bzw. nicht in dieser Kombination ausüben.

Die Einfuhr von SoHO in die EU oder die Ausfuhr von SoHO aus der EU sind ebenfalls Tätigkeiten, die eine SoHO-Betriebsstätte definieren, dieser Aspekt wird in dieser Übersicht jedoch nicht weiter ausgeführt.

Eine beispielhafte und nicht erschöpfende Übersicht über die sich aus dem Anwendungsbereich der Regulierung ergebende Einstufung von Humanmilch verarbeitenden Einrichtungen ist in Tab. 2 aufgeführt.

**Table:** **Tab. 2** Einstufung von Humanmilch verarbeitenden Einrichtungen und definierende Eigenschaft nach Verordnung (EU) 2024/1938.

Einrichtung	Definierende Eigenschaft	Einstufung
Frauenmilchbank	Verarbeitung *und* Lagerung, Freigabe	Betriebsstätte
Abteilung pasteurisiert Muttermilch	Verarbeitung *und* Lagerung, Freigabe	Betriebsstätte (bei Ausnutzung des Umsetzungsspielraums durch den Gesetzgeber: Einrichtung)
Abteilung mit Bezug von Frauenmilch aus einer externen Frauenmilchbank ^ a^	Lagerung, Verwendung beim Menschen	Einrichtung
Abteilung rekrutiert Spenderinnen für eine externe Frauenmilchbank ^b^	Überprüfung der Anamnese von SoHO-Spenderinnen, Gewinnung, Lagerung	Einrichtung
Abteilung ohne Frauenmilch und ohne Pasteurisierung von Muttermilch	entfällt	Keine Anwendung

^a^ Dies gilt unabhängig von der Bezugsform (gefrorene oder gefriergetrocknete Frauenmilch) und von der Bezugsquelle (nichtkommerzielle Frauenmilchbank, kommerzieller Anbieter); ^b^ Sofern die Verarbeitung und Freigabe der gewonnenen Frauenmilch in der externen Frauenmilchbank erfolgt.

Sollten in einem Krankenhaus oder einem Klinikverbund mehrere SoHO-Einrichtungen betrieben werden, ist eine übergreifende Registrierung nur in dem Fall vorgesehen, dass dieselbe verantwortliche Person für alle Einrichtungen benannt wurde und ein übergreifendes Qualitätsmanagementsystem besteht.

## Organisatorische Pflichten für SoHO-Einrichtungen und SoHO-Betriebsstätten

Die Pflichten für SoHO-Einrichtungen und SoHO-Betriebsstätten sind in Tab. 3 dargestellt. Während einige der in der Verordnung aufgeführten Pflichten sich bereits aus der Regulierung als Lebensmittel ergaben, kommen unter der neuen SoHO-Regulierung weitere Anforderungen an SoHO-Einrichtungen hinzu. Die SoHO-Betriebsstätten unterliegen darüber hinaus zusätzlichen Pflichten.

**Table:** **Tab. 3** Pflichten von SoHO-Einrichtungen und -Betriebsstätten.

**Pflichten für SoHO-Einrichtungen**	
Registrierungspflicht	Digitale Registrierung auf der SoHO-Plattform der EU generiert eine SoHO-Identifizierungsnummer für die Einrichtung
Benennung einer verantwortlichen Person	
Einrichtung eines Rückverfolgungs-, Kodierungs- und Vigilanzsystems	Vigilanzmeldungen bei schwerwiegenden unerwünschten Reaktionen und Zwischenfällen an zuständige Behörde
Erhebung und Meldung von Tätigkeitsdaten	Meldung über SoHO-Plattform der EU
**Zusätzliche Pflichten für SoHO-Betriebsstätten**	
Erlaubnis als SoHO-Betriebsstätte	Erlaubnis durch Landesbehörden als Voraussetzung für die Ausübung von SoHO-Tätigkeiten
Regelmäßige Inspektion durch Landesbehörden	Maximales Inspektionsintervall 4 Jahre
Benennung von Freigabeverantwortlichen	Bei der verantwortlichen, freigabeverantwortlichen und ärztlichen Person kann es sich um eine einzelne Person handeln, die alle Funktionen wahrnimmt, sofern die jeweiligen Voraussetzungen erfüllt sind.
Benennung einer ärztlichen Person	

EU, Europäische Union; SoHO, *substances of human origin*

## Zulassungspflicht für SoHO-Präparate

Ein zentrales neues Element im Humanmilchbereich ist die Zulassungspflicht für alle SoHO-Präparate. SoHO-Präparate sind definiert als SoHO, die einer Verarbeitung unterzogen wurden, eine spezifische klinische Indikation aufweisen und zur Verwendung beim Menschen in einem bestimmten Patientenkollektiv oder zur Verteilung bestimmt sind.

Eine Zulassung für ein SoHO-Präparat wird von der zuständigen Behörde erteilt, sofern eine positive Nutzen-Risiko-Bewertung auf Grundlage wissenschaftlicher Nachweise und klinischer Daten vorliegt. Bei unzureichenden wissenschaftlichen Nachweisen und klinischen Daten für eine positive Nutzen-Risiko-Bewertung muss von den SoHO-Einrichtungen ein Plan für die Überwachung klinischer Ergebnisse eingereicht werden, der Grundlage für die weitere Datenerhebung ist und dessen Anforderungen sich nach dem Risikoprofil des SoHO-Präparats richten. Die EU-Kommission wird für die Ermittlung des Risikos ein Online-Tool zur Verfügung stellen, mit dessen Hilfe die Einrichtungen das Risikoprofil des SoHO-Präparats ermitteln können. Die finale Entscheidung über Art und Umfang der weiteren Datenerhebung wird jedoch von der für die Zulassungen zuständigen Behörde getroffen. Als zuständige Bundesoberbehörde u. a. für die Zulassung von Blut- und Gewebezubereitungen wird das Paul-Ehrlich-Institut (PEI) auch die Zuständigkeit für die Zulassung von Humanmilchpräparaten übernehmen.

Aufgrund der langjährigen Verwendung und umfassenden Erfahrungen mit Humanmilch ist es möglich, dass sie der niedrigsten Risikogruppe zugeordnet wird oder bereits genügend wissenschaftliche Erkenntnisse vorliegen, die eine Zulassung ohne zusätzliche Auflagen rechtfertigen.

Ausgenommen von der Definition der Verarbeitung, mit der Folge, dass keine Zulassungspflicht für das SoHO begründet wird, sind erforderliche Verfahren unmittelbar vor der Verwendung des bereits freigegebenen SoHO, z. B. das Erwärmen der Milch im Flaschenwärmer oder das Aufziehen einzelner Mahlzeiten aus sog. Milchsammelflaschen. Auch das Vermischen von humaner Milch mit Arzneimitteln oder Nahrungsergänzungsmitteln (Elektrolyte, Mineralien, bovine Humanmilchverstärker) vor der Verabreichung fällt nicht unter die Definition der Verarbeitung.

## Ausführungsbestimmungen

Die Standards für den Umgang mit humaner Milch, welche den SoHO-Einrichtungen als Ausführungsbestimmungen und den Aufsichtsbehörden als Grundlage ihrer Inspektionen dienen werden, sind im *Guide to the quality and safety of tissues and cells for human application* des Europäischen Direktorats für die Qualität von Arzneimitteln und Gesundheitsfürsorge des Europarats (EDQM) niedergelegt (Tissue&Cell-Guide). In der für Ende 2026 zur Veröffentlichung geplanten 6. Auflage des Tissue&Cell-Guides wird Humanmilch erstmals als SoHO aufgeführt. Die Entwurfsfassung wurde im Oktober 2025 fertiggestellt und befindet sich momentan in der Konsultationsphase mit den verschiedenen Interessenvertretern.

Der Tissue&Cell-Guide beinhaltet im ersten Abschnitt (Teil A) allgemeine Kapitel, die übergreifende Gültigkeit für alle SoHO besitzen und unter anderem Bestimmungen zu Qualitäts- und Risikomanagement, Spenderinnenrekrutierung und -testung, räumlichen und organisatorischen Voraussetzungen von SoHO-Einrichtungen sowie Bestimmungen zur Verarbeitung, Verpackung, Nachverfolgbarkeit und Lagerung von SoHO. Die gewebespezifischen Kapitel im Teil B, darunter das Humanmilchkapitel, enthalten an das jeweilige SoHO angepasste Erläuterungen und Bestimmungen.

## Unterstützende Maßnahmen zur europäischen Harmonisierung

Mehrere parallele europäische Initiativen und Verbundprojekte sollen zum Prozess der europäischen Harmonisierung beitragen. Im Folgenden werden die für das Humanmilchwesen relevantesten Initiativen dargestellt.

Konsortialprojekt IMAGINE-HMB

Das von der Europäischen Union geförderte Konsortialprojekt „Implementation of human Milk harmonized Guidelines for Infant Nutrition in Europe – Human Milk Banking“ (IMAGINE-HMB) wird von der European Milk Banking Association (EMBA) geleitet 9 . Gemeinsam mit den Frauenmilchbankgesellschaften aus Polen, Spanien und der deutschen Frauenmilchbank-Initiative e.V. (FMBI) sowie einer irischen Elternorganisation erarbeitet das Projekt europäische Konsensus-Leitlinien für den Betrieb von Frauenmilchbanken. Diese Leitlinien sollen als fachliche Ergänzung des technischen Tissue&Cell-Guide dienen, müssen diesem im Grundsatz jedoch inhaltlich entsprechen. Die Fertigstellung der Leitlinien ist für Anfang 2026 vorgesehen. Perspektivisch sollen die Inhalte dieser Leitlinien in einer Neuauflage des Tissue&Cell-Guide übernommen werden.

Leitlinien des European Centre for Disease Prevention and Control

Das European Centre for Disease Prevention and Control (ECDC) erarbeitet technische Leitlinien zur Prävention der spenderassoziierten Übertragung von Erkrankungen durch SoHO 10 . Eine erste Leitlinie für die HIV-Übertragung ist im September 2025 publiziert worden, eine dezidierte Richtlinie für das Screening humaner Milch ist in Planung, wird aber nicht vor 2027 publiziert werden. Die ECDC-Leitlinien sind von den SoHO-Einrichtungen verbindlich zu berücksichtigen. Perspektivisch sollen sie Bestandteil des Tissue&Cell-Guides werden.

GAPP-PRO

Das EU-geförderte Verbundprojekt “FacilitatinG the Authorisation of Preparation Process for blood and tissues and cells – Piloting GAPP model approach for assessing and authorizing novel substances of human origin preparation PROcesses” (GAPP-PRO, 2024-2027) adressiert die Harmonisierung in der Bewertung und Zulassung von SoHO-Präparaten unter den Mitgliedstaaten durch Erstellung entsprechender technischer Leitlinien 11 . Das Projekt soll eine konsistente und effiziente Implementierung der neuen Regulierung in den Mitgliedstaaten unterstützen. Ein Hauptziel ist die Testung und Weiterentwicklung eines einheitlichen Zulassungsdossiers (preparation processing dossiers (PPD)), das als harmonisierter Ansatz für die Einrichtung von SoHO-Zulassungen auf der SoHO-Plattform genutzt werden soll.

Vigilanz-Arbeitsgruppe des SoHO-Koordinierungsgremiums

Die Vigilanz-Arbeitsgruppe operiert als Subgruppe des SoHO-Koordinationsgremium, das von der EU-Kommission zur Unterstützung der Mitgliedstaaten bei der Umsetzung der SoHO-Verordnung gegründet wurde 12 . Diese Arbeitsgruppe entwickelt spezifische Vigilanzprotokolle und -prozesse u. a. für Humanmilch, die in das übergeordnete EU-Vigilanzsystem integriert werden sollen.

## Nationale Umsetzung in Deutschland

Das Ziel des deutschen Umsetzungsprozesses besteht darin, die hohen Qualitäts- und Sicherheitsstandards zu erhalten, die Versorgungssicherheit weiter zu verbessern und dabei die administrative Belastung möglichst gering zu halten.

Die Mitgliedstaaten haben für verschiedene Bereiche der SoHO-Verordnung unterschiedliche Übergangsfristen. Für den Bereich Humanmilch gelten die in **Infobox 1** dargestellten Fristen. Die genannten Fristen setzen teilweise die zeitgerechte Funktionsfähigkeit der SoHO-Plattform voraus.

Das Bundesministerium für Gesundheit erarbeitet derzeit einen Gesetzesentwurf zur Durchführung der SoHO-Verordnung. Das Konzept sieht ein Gesetz vor, das alle SoHO unter einen gemeinsamen Rechtsrahmen stellt und dabei sowohl die Zulassungen für SoHO-Präparate, Registrierungen der SoHO-Einrichtungen und Erlaubnisse für SoHO-Betriebsstätten, Inspektionen, Spender- und Empfängerschutzbestimmungen, Vigilanz, Meldung von Tätigkeitsdaten als auch die Maßnahmen zur Sicherstellung der Versorgung umfasst.

Der Übergang in das offizielle Gesetzgebungsverfahren ist für das Frühjahr 2026 vorgesehen.

## Herausforderungen und offene Fragen

Die Implementierung der SoHO-Verordnung stellt den Gesetzgeber, die Behörden und SoHO-Einrichtungen vor Herausforderungen.

Registrierung und Betriebserlaubnis

Bei der Registrierung der SoHO-Einrichtungen handelt es sich um eine neue Pflicht für alle SoHO-Einrichtungen. Sie soll aufwandsarm über die neue SoHO-Plattform der EU erfolgen. Die Registrierung wird von der zuständigen Landesbehörde geprüft und freigegeben.

Frauenmilchbanken werden darüber hinaus eine Erlaubnis als SoHO-Betriebsstätte der zuständigen Landesbehörde benötigen. Entscheidendes Merkmal der Betriebsstätte ist die Kombination der Tätigkeiten „Verarbeitung und Lagerung“ oder der Freigabe von Frauenmilchpräparaten (siehe auch **Infobox 2** ).

Die Erlaubnispflicht gilt wiederum für alle neonatologischen Abteilungen, die Humanmilch verarbeiten, insbesondere pasteurisieren, unabhängig davon, ob diese Milch für das eigene Kind (Muttermilch) oder für fremde Kinder (Frauenmilch) gedacht ist. Der nationale Gesetzgeber hat jedoch insoweit Umsetzungsspielraum, dass er festlegen kann, dass die Muttermilch nur im Fall der Verarbeitung durch bestimmte spezialisierte Einrichtungen von der Verordnung umfasst ist. Es wäre demnach denkbar, dass neonatologische Abteilungen, die ausschließlich Muttermilch pasteurisieren, jedoch keine Frauenmilch herstellen, nicht als SoHO-Betriebsstätte klassifiziert werden und demnach keine Erlaubnis, sondern ggf. nur eine Registrierung benötigen.

Eine abschließende Klärung, welchem rechtlichen Rahmen humane Muttermilchverstärkerkünftig zuzuordnen sein werden, steht derzeit noch aus.

### Digitalisierung des Humanmilchwesens

Die Digitalisierung des Humanmilchwesens wird mittelfristig empfehlenswert, um den wachsenden Anforderungen an Kennzeichnung, Lagerverwaltung, Rückverfolgbarkeit und jährliche Berichterstattung in Bezug auf SoHO gerecht zu werden.

Die vollständige Implementierung digitaler Systeme stellt die betreibenden Kliniken jedoch vor erhebliche finanzielle und organisatorische Herausforderungen. Derzeit sind in Deutschland keine SoHO-konformen digitalen Verwaltungsprogramme, die speziell auf die Anforderungen des Humanmilchwesens zugeschnitten sind, in Anwendung. Einige Einrichtungen nutzen angepasste Versionen kommerzieller Verwaltungsprogramme, die ursprünglich für Blut- oder Gewebebanken entwickelt wurden.

### Vigilanzsystem für unerwünschte Reaktionen und Zwischenfälle

Alle SoHO-Anwendungen bedürfen organisierter Überwachungs- und Meldeverfahren in Bezug auf unerwünschte Reaktionen bei den Humanmilch-Empfängern und Zwischenfällen. Leitlinien zur Vigilanz im Bereich der Humanmilch liegen derzeit nicht vor, eine Erarbeitung auf europäischer Ebene ist unter Einbezug von Vertretern der EMBA jedoch geplant. Die fachliche neonatologische Expertise ist hierbei essentiell, da es sich bei dem Empfängerkollektiv grundsätzlich um instabile Frühgeborene mit einer hohen Krankheitslast und Letalität handelt. Es müssen realistische und anwendbare Kriterien für schwerwiegende unerwünschte Reaktionen bei den Frühgeborenen durch eine Humanmilchgabe definiert werden.

Finanzierung Eine finanzielle Unterstützung der Länder bei den notwendigen Struktur- und Prozessanpassungen der Frauenmilchbanken ist nach jetzigem Kenntnisstand nicht vorgesehen. In der Vergangenheit wurde in Niedersachsen, Schleswig-Holstein und Nordrhein-Westfalen der Aufbau von Frauenmilchbanken aus Haushaltsmitteln der Länder unterstützt (siehe Infobox 3).

**Infobox 3**

Die Frauenmilchbank-Initiative e.V. empfiehlt den Trägern von Frauenmilchbanken, das Gespräch mit den jeweils landespolitisch Verantwortlichen zu suchen, um eine gezielte Sonderförderung – beispielsweise zur Digitalisierung der Frauenmilchbanken – in die kommenden Haushaltsplanungen der Bundesländer einzubringen. Ein entsprechendes Fördervorhaben ist beispielsweise in Nordrhein-Westfalen bereits in Planung.

### Praktische Arbeitsweisen in Frauenmilchbanken

Die praktische Arbeitsweise von Frauenmilchbanken in Deutschland wird bisher durch eine umfassende S2k-Leitlinie geregelt 8 . Diese Leitlinie trug in ihrer Konzeption der mangelnden Evidenz für viele Vorgehensweisen in der Behandlung humaner Milch Rechnung und reflektierte die sehr unterschiedlichen praktischen Arbeitsweisen in den Frauenmilchbanken. Das Ausmaß der Auswirkung der SoHO-Verordnung, bzw. des Tissue&Cell-Guide auf die Arbeitsweise von Frauenmilchbanken ist aufgrund des laufenden Konsultationsverfahrens und der ausstehenden Harmonisierung mit der IMAGINE-HMB-Leitlinie noch nicht abschließend zu beurteilen. Erschwert wird die europäische Harmonisierung durch die Vielzahl an unterschiedlichen Frauenmilchbanken-Modellen und unterschiedlichen nationalen Ressourcen im Frauenmilchbankwesen. Grundsätzlich besteht die Gefahr, dass zu hohe Anforderungen den Betrieb von Frauenmilchbanken und die Verfügbarkeit von Frauenmilch einschränken. Dies könnte zu einer Verschlechterung der medizinischen Versorgung von Frühgeborenen führen und sollte im Rahmen des Umsetzungsverfahrens durch den Gesetzgeber entsprechend berücksichtigt werden.

## Beteiligung von Fachgesellschaften

Die erfolgreiche nationale Umsetzung der SoHO-Verordnung im Humanmilchbereich erfordert die aktive Beteiligung von sachkundigen Fachgesellschaften. Nach ersten Gesprächen unter Beteiligung von Vertretern des Bundesministeriums für Gesundheit, des PEI, der Gesellschaft für Neonatologie und Pädiatrische Intensivmedizin (GNPI) und der FMBI sollen sachkundige Gesellschaften, darunter die GNPI, die FMBI und die Deutsche Krankenhaus Gesellschaft in das Anhörungsverfahren zum Gesetzesentwurf einbezogen werden. Diese Beteiligung ist essentiell, um praxisrelevante Aspekte zu identifizieren und eine sachgerechte Umsetzung zu gewährleisten.

## Zusammenfassung und Ausblick

Die Verordnung (EU) 2024/1938 markiert einen fundamentalen Wandel in der rechtlichen Behandlung humaner Milch in Europa. Mit dem Inkrafttreten am 6. August 2024 und gestaffelten Umsetzungsfristen bis August 2028 werden gespendete Frauenmilch und verarbeitete (pasteurisierte) Humanmilch aus dem Lebensmittelrecht herausgelöst und als SoHO mit entsprechenden Qualitäts- und Sicherheitsstandards reguliert.

Für Deutschland bedeutet dies zwar eine grundsätzliche Kompatibilität mit dem bestehenden System, jedoch auch die Notwendigkeit, neue Elemente wie die flächendeckende Registrierung aller SoHO-Einrichtungen, die Zulassungspflicht für alle SoHO-Präparate und erweiterte Vigilanzverpflichtungen zu implementieren. Frauenmilchbanken und neonatologische Abteilungen, die Muttermilch pasteurisieren, werden als SoHO-Betriebsstätten klassifiziert und unterliegen erhöhten Anforderungen hinsichtlich Organisation, Personal und behördlicher Überwachung.

Die parallelen europäischen Harmonisierungsbemühungen schaffen die erforderliche fachliche Grundlage für eine konsistente Umsetzung der Verordnung. Insbesondere die inhaltlichen Ausgestaltungen und die rechtzeitige Verfügbarkeit des Tissue&Cell-Guide (6. Edition, 2026) und der IMAGINE-HMB-Guidelines (2026) wird für die Praxis von entscheidender Bedeutung sein.

Der derzeit in Erarbeitung befindliche deutsche Gesetzesentwurf muss die Balance zwischen der Gewährleistung hoher Qualitäts- und Sicherheitsstandards, der Praktikabilität für die betroffenen Einrichtungen und der Sicherstellung einer flächendeckenden Versorgung mit humaner Milch finden. Die dreijährige Übergangsfrist bietet den Einrichtungen die Möglichkeit, sich schrittweise auf die neuen Anforderungen vorzubereiten (siehe Infobox 4).

**Infobox 4**

Die Frauenmilchbank-Initiative e.V. wird den Umsetzungsprozess in den kommenden Jahren weiterhin aktiv begleiten und ihre Mitglieder durch Webinare, Fristenerinnerungen sowie praxisnahe Handlungsempfehlungen gezielt bei der Umsetzung der SoHO-Verordnung unterstützen.

Die SoHO-Verordnung bietet trotz der Herausforderungen die Chance, die Versorgung mit humaner Milch auf eine stabilere rechtliche und qualitative Basis zu stellen und durch Vereinheitlichung der Qualitäts- und Sicherheitsstandards in der EU den Zugang zu dieser essentiellen Ressource für vulnerable Frühgeborene zu verbessern. Der Erfolg wird maßgeblich davon abhängen, ob die notwendigen Ressourcen für Infrastruktur, Personal und Schulungen bereitgestellt werden und ob die Einbindung der Fachgesellschaften in den Umsetzungsprozess zu praxisgerechten Regelungen führt.

## Figures and Tables

**Table:** 

Umsetzungsfristen der SoHO-Verordnung	
7. August 2027	Registrierung der Einrichtungen, Einhaltung der Spender- und Empfängerschutzbestimmungen
9. November 2027	Beantragung von Zulassungen für SoHO-Präparate; Beantragung von Erlaubnissen für SoHO-Betriebsstätten
9. August 2028	Anwendung der restlichen Bestimmungen der SoHO-Verordnung

## Notes

Infobox 1

## Notes

Infobox 2 Die Unterscheidung zwischen SoHO-Einrichtung und SoHO-Betriebsstätte ist insbesondere für Kliniken relevant, die im Rahmen eines Netzwerkes oder einer Kooperation Frauenmilch an andere neonatologische Abteilungen abgeben bzw. zu verarbeitende Milch von diesen empfangen. Hier gelten die zentralen Frauenmilchbanken, die in einer Kooperation oder in einem Netzwerk die zentralen Aufgaben der Frauenmilchverarbeitung – insbesondere die Verarbeitung und Lagerung sowie Freigabe – übernehmen, als Betriebsstätten. Neonatologische Abteilungen, die innerhalb eines Frauenmilchbank-Netzwerks nicht die zentrale Aufgabe der Frauenmilchverarbeitung erfüllen, werden hingegen nicht als Betriebsstätten, sondern als SoHO-Einrichtungen eingestuft. Dies bedeutet, dass in diesen Kliniken Tätigkeiten der SoHO-Produktkette – wie beispielsweise die Rekrutierung von Spenderinnen, das Sammeln, Lagern, Versenden, Empfangen und Ausgeben von Frauenmilch – weiterhin durchgeführt werden können. Eine entsprechende Kooperation muss vertraglich vereinbart sein.
